# Modified Inguinal Microscope-Assisted Varicocelectomy under Local Anesthesia: A Non-randomised Controlled Study of 3565 Cases

**DOI:** 10.1038/s41598-018-21313-0

**Published:** 2018-02-12

**Authors:** Jin Wang, Qian Liu, Xun Wang, Rijian Guan, Sen Li, Youpeng Zhang, Yongbiao Cheng, Hanqing Zeng, Yong Tang, Zhaohui Zhu

**Affiliations:** 1 Department of Urology, Union Hospital, Tongji Medical College, Huazhong University of Science and Technology, Wuhan, 430022 China; 2 Department of Nosocomial Infection Management, Union Hospital, Tongji Medical College, Huazhong University of Science and Technology, Wuhan, 430022 China

## Abstract

Varicocele is a common abnormality, but the conventional microsurgical subinguinal varicocelectomy (CMSV) has some disadvantages. We invented Modified Inguinal Microscope-Assisted Varicocelectomy (MIMV) under local anesthesia. This study aims to evaluate MIMV by comparing it to CMSV in operating duration, time to return to normal activity, postoperative complications, achievement of natural pregnancy and improvement of semen quality for patients with infertility, pain score for those with scrotal pain, and so on. We enrolled 3089 patients who underwent MIMV and 476 who underwent CMSV in our hospital. Both the operating duration and the time to return to normal activity of MIMV was shorter than that of CMSV (P < 0.001). The recurrence rate (P < 0.001) and injury rate of vas deferens (P = 0.011) after MIMV were lower than that after CMSV. Moreover, patients with MIMV showed higher degree of satisfaction with the surgery experience and outcome than those with CMSV (P < 0.001). However, no statistical difference was found between the two groups in scores of pain due to surgery, postoperative varicose veins diameters, reflux duration, and the postoperative complications of wound infection, hydrocele, atrophy of testis, epididymitis, and scrotal hematoma. In summary, MIMV is a promising varicocelectomy and could be applied more in clinical practice.

## Introduction

Varicocele is a common abnormality of the testis, characterized by excessive dilatation of the pampiniform venous plexus of the spermatic cord^1^. The andrological implications of varicocele include failure of ipsilateral testicular growth and development, symptoms of pain and discomfort, and male infertility^2^. The main goal of all varicocele treatment is to resolve these problems^3,4^. The aim of treatment in children is to prevent testicular injury and maintain normal testicular function, which can be achieved by surgical ligation of varicoceles^5,6^.

Many clinical trials report a beneficial effect of varicocele repair on male reproduction with improvement in semen parameters, whereas other studies suggest the contrary^7^. Several methods for varicocele repair have been used, including the traditional inguinal or high retroperitoneal approaches, laparoscopic repair and microsurgical varicocelectomy via an inguinal or subinguinal incision^8^. Each technique has advantages and disadvantages, and conflicting results have been reported by different studies^9^. Laparoscopic varicocelectomy is associated with lower postoperative pain scores, less time to return to normal activity and higher patient satisfaction compared with conventional transperitoneal varicocelectomy^10^. In addition, subinguinal microsurgery has less complications, but the operating duration was significantly longer in the microscopic group in comparison with open inguinal technique and laparoscopy^9^.

Thus, we invented Modified Inguinal Microscope-Assisted Varicocelectomy (MIMV) under local anesthesia based on open inguinal varicocelectomy and microsurgical varicocelectomy. MIMV represents an evolution of varicocelectomy and aims to reduce surgical trauma, operating duration, recovery time and post-operative complications.

In this study, we present our experience of spermatic cord ligation with MIMV technique and compare it with the conventional microscope-assisted subinguinal varicocelectomy (CMSV).

## Methods

From September 2008 to March 2016, we enrolled patients who were diagnosed with clinical palpable unilateral or bilateral varicocele and underwent MIMV in our hospital. According to the European Association of Urology (EAU) Guideline^11^, clinical palpable varicocele has been classified into Grade 1 (palpable during Valsava manoeuvre, but not otherwise), Grade 2 (palpable at rest, but not visible) and Grade 3 (visible and palpable at rest).

According to the EAU Guideline and Guideline for Diagnosis and Treatment of Urological Disease in China (2014), the indications of varicocelectomy were: (1) infertility because of low semen quality; (2) scrotal pain; (3) persistent prostatitis with clinical palpable varicocele; (4) no symptoms, but varicocele in Grade 3 was found in medical examination (entrance examination for some special professions in China, such as pilots and soldiers); (5) testicular atrophy in adolescents^11^. When patients had two or more indications, we chose the first one for description.

The option of MIMV and CMSV was offered to every patient, and the choice of method was made by the patients themselves. All the patients underwent a complete physical examination before surgery, including supine and standing scrotal examination and a color Doppler ultrasound examination. Doppler ultrasound was performed to confirm the diagnosis and to measure the diameters of varicose veins and duration of reflux^12^.

The surgical procedures of MIMV were performed as follows:

1. The incision location was marked (A. surface projection of external inguinal ring, B. a 2-cm marking line at the direction of iliac crest with 1 cm away from the external inguinal ring (① at the 2/5 outside of the vertical distance between ipsilateral anterior superior iliac spine and median line of abdomen, ② the upper margin of incision, ③ the lower margin of incision, ④ the midpoint of the incision) (Supplementary Figure 1). Local anesthetic solution was used (20 ml of 2% lidocaine combined with 20 ml normal saline and 0.1 ml of 0.1% adrenaline).
2. A combination of local anesthesia and IV sedation^13^ was used for the patients who were either overweight (BMI ≥ 30), very nervous, or requested IV sedation.
3. Conventional disinfection and drape surgical towels were applied with the patient placed in supine position. The needle was injected at site ① and stopped when it passed through the abdominal external oblique aponeurosis with an obviously falling feeling. Subsequently, 9 ml of local anesthetic were injected to block the iliohypogastric nerve. The needle was injected at site ② and stopped as the same as site ①, and 9 ml of local anesthetic were injected, infiltrated layer by layer through the abdominal external oblique aponeurosis, the subcutaneous tissue and the ilioinguinal nerve. Cutaneous and subcutaneous infiltration anesthesia was performed at site ③, and 6 ml of local anesthetic were injected, infiltrated layer by layer to block subcutaneous tissue incision. Cutaneous and subcutaneous infiltration anesthesia was also performed at site ④, with 3 ml of local anesthetic injected, infiltrated layer by layer to block the skin and subcutaneous tissue of the incision.
4. The skin and subcutaneous tissue were incised, exposing the external oblique aponeurosis, and a longitudinal incision was made. Blunt dissection of the cremaster muscle was performed and spermatic cord below the muscle was identified, facilitating the placement of the spermatic cord to the skin incision with a piece of rubber sheet (Supplementary Figure 2).
5. The spermatic vascular bundle was then placed under a microscope with a resolution increase of 8-fold. The fascia of the spermatic cord was incised, and 1–2 larger spermatic veins were raised, to expose the remaining spermatic cord blood vessels and surrounding fat tissue which were raised at the same time, visualizing the obvious boundaries with the vas deferens vascular system.
6. Blunt dissection at the junction of the vas deferens vascular system was conducted (Supplementary Figure 3).
7. The testicular artery and lymph vessels were carefully isolated. Vascular pulsation and blood flashing were observed or micro ultrasonic Doppler was used to confirm and protect the testicular artery. All the internal spermatic veins were ligated using Surgical Silk 5–0 and incised. Thick veins with no artery or lymph-vessel around could be ligated together (Supplementary Figure 4).
8. Finally, a check was performed for obvious leakage and bleeding, sutured the fascia of the spermatic cord and the muscle of the testis, sutured each layer, and glued the incision (Supplementary Figure 5).

CMSV was performed as previously described^14,15^. Lumbar anesthesia was used for most cases of CMSV^16^, and general anesthesia was performed for patients who were very nervous or had lumbar lesions. Eleven vessels on average were ligated for each patient of CMSV^17^.

All the procedures of MIMV and CMSV were performed by the same surgeons. Postoperative analgesia was not performed. Patients were told that the wound cannot touch water for a week and strenuous exercise is forbidden for a month. The operating duration (excluding anesthesia), time to return to normal activity and postoperative complications of every patient were recorded. Time to return to normal activity was calculated by the time needed to perform the activities of daily living (such as feeding ourselves, bathing, dressing, grooming, work, homemaking, and leisure; not including lifting and extraneous exercise) with pain^10^. The surgical complications were reported using the Clavien Classification of Surgical Complications^18^. Postoperative pain was assessed at postoperative hours 3, 24 and 48 using visual analogue scale (VAS) pain scores ranging from 0 (no pain) to 10 (worst pain). Patients were instructed to complete the rest of VAS forms at home and return them 7 days after the surgery. Doppler ultrasound was performed at 1–3 months postoperatively to ensure complete ligation of the varicocele^19^, and it was also performed at six months postoperatively to assess testicular size and recurrence^20^. Varicocele recurrence was considered to be present if retrograde flow for at least 2 s was identified within the pampiniform plexus spontaneously and/or during deep inspiration^21^. We tried to perform postoperative Doppler ultrasound on every subject, and patients who did not attend outpatient appointments were excluded from the study. Patient satisfaction regarding the cosmetic outcome was assessed at 3 months postoperatively by asking the patients to rate their level of satisfaction by phone as either very satisfied, somewhat satisfied, somewhat dissatisfied, or very dissatisfied.

For the patients with infertility, two semen samples were collected by masturbation within one week, preoperatively and 90 days after MIMV^22^. Semen samples were obtained by masturbation after 5 days of sexual abstinence, and the analysis was conducted according to the WHO laboratory manual for the Examination and processing of human semen (5^th^ edition)^23^. The 2010 WHO reference values were a semen volume >1.5 mL, sperm concentration >15 million/mL, total motility (A + B + C) > 40%, and normal morphology >4% by Kruger criteria. For comparison, only concentration and motility criteria were used. Patients showing sperm concentration and motility data above the reference values were classified as normal. Patients with a concentration or motility below the reference values were determined as having suboptimal sperm quality^24^. An improved seminal result after varicocelectomy was defined as a ≥20% increase in sperm concentration or motility^9,24–26^. The natural pregnancy within 12 months after the surgery was also recorded.

For patients with scrotal pain, the pain due to disease was assessed before and one month after the operation using the VAS scale.

For patients with persistent prostatitis, symptoms were assessed with before and one month after the operation using the Chronic Prostatitis Symptom Index of the National Institutes of Health (NIH-CPSI)^27^.

For patients with no symptoms and adolescents with testicular atrophy, physical examination was performed before and one month after the operation.

### Statistical analysis

We analyzed the data with the Statistical Package for the Social Sciences, Version 11.0 (SPSS, Chicago, IL). Age, operating duration, scores of pain due to surgery, and time to return to normal activity were compared between MIMV and CMSV by t-test. The indications of varicocelectomy, laterality, Grade, postoperative complications, satisfaction, natural pregnancy, and semen results were compared between MIMV and CMSV by chi square test. The sperm concentration, sperm motility and scores of pain due to disease were compared before and after MIMV using paired t-test. P < 0.05 was considered to be statistically significant.

### Ethical consideration

Informed consent was obtained from all participants and confidentiality was ensured. The study was approved by the ethics committee of Tongji Medical College, Huazhong University of Science and Technology. Our methods were performed in accordance with the European Association of Urology (EAU) Guidelines, and performing varicocelectomy for patients with persistent prostatitis and clinical palpable varicocele conformed to the Guideline for Diagnosis and Treatment of Urological Disease in China.

### Data availability

The datasets generated during and/or analyzed during the current study are available from the corresponding author on reasonable request.

## Results

In total, 3324 patients underwent MIMV and 565 patients underwent CMSV. 235 (7.1%) patients with MIMV and 89 (15.8%) patients with CMSV were lost in follow up after surgery and were excluded from the study. Consequently, 3089 patients with MIMV and 476 patients with CMSV were included in the study. IV sedation was used in 0.9% (29/3089) of the patients with MIMV.

No statistical difference was found in age, indications of varicocelectomy, laterality, grade, preoperative diameters of varicose veins, and preoperative duration of reflux between MIMV patients and CMSV patients (Table 1).

**Table 1:** Characteristics of all the patients in two groups.

Characteristic	MIMV	CMSV	t/X^2^	P value
Age, year				
Mean ± SD	28.6 ± 9.3	29.5 ± 8.7	0.798	0.431
Range	11–82	13–76		
Preoperative diameters of varicose veins, mm	3.2 ± 0.6	3.3 ± 0.7	0.976	0.324
Preoperative reflux duration, sc	4.5 ± 2.4	4.4 ± 2.3	0.942	0.386
Indications of varicocelectomy, n (%)				
Infertility because of low semen quality	2094 (67.8)	330 (69.3)		
Scrotal pain	577 (18.7)	79 (16.6)		
Persistent prostatitis with clinical palpable varicocele	320 (10.4)	56 (11.8)	2.896	0.575
With no symptoms, but varicocele in Grade 3 was found in examination	61 (2.0)	7 (1.5)		
Testicular atrophy in adolescent	37 (1.2)	4 (0.8)		
Varicocele laterality, n (%)				
Unilateral	1921 (62.2)	312 (65.5)	1.987	0.159
Bilateral	1168 (37.8)	164 (34.5)		
Varicocele grade, n (%)				
Grade 1	1890 (61.2)	280 (58.9)		
Grade 2	519 (16.8)	83 (17.5)	1.029	0.598
Grade 3	680 (22.0)	113 (23.7)		

As Table 2 shows, the operating duration was shorter for both Unilateral MIMV (t = 3.500, P < 0.001) and bilateral MIMV (t = 4.456, P < 0.001) compared with CMMV. The time to return to normal activity was less with MIMV than with CMSV (t = 9.014, P < 0.001). The rates of both recurrence and injury of vas deferens after MIMV were lower than that after CMSV (X^2^ = 18.460, P < 0.001; X^2^ = 6.51, P = 0.011, respectively). The patients with MIMV were more satisfied that those with CMSV (X^2^ = 33.045, P < 0.001). There was no difference between the two groups in time to follow up, scores of pain due to surgery, varicose veins diameters at 6 months postoperatively, reflux duration at 6 months postoperatively, and other postoperative complications. Complete ligation of varicocele was found in all the patients of the two groups.

**Table 2:** Operative and postoperative outcomes of all the patients in two groups.

Outcomes	MIMV	CMSV	t/X^2^	P value
Operating duration, min				
Unilateral	20.2 ± 2.1 (17–25)	40.2 ± 4.1 (35–30)	325.500	<0.001*
Bilateral	37.0 ± 3.5 (32–42)	80.0 ± 9.1 (70–95)	312.456	<0.001*
Scores of pain due to surgery				
At 3 h	4.3 ± 1.7 (1–9)	4.5 ± 1.2 (2–9)	0.735	0.467
At 24 h	3.4 ± 1.5 (0–7)	3.2 ± 1.8 (1–8)	0.958	0.361
At 48 h	2.5 ± 1.6 (0–6)	2.6 ± 1.3 (0–8)	0.697	0.511
Time to return to normal activity, hour	12.8 ± 6.3 (6–24)	25.9 ± 9.5 (16–48)	9.014	<0.001*
Varicose veins diameters at 6 months postoperatively, mm	1.6 ± 0.2	1.7 ± 0.1	0.965	0.356
Reflux duration at 6 months postoperatively, sc	0.3 ± 0.1	0.3 ± 0.2	0.996	0.314
Time to follow up, months				
Mean ± SD	12.1 ± 6.3	12.4 ± 5.9	1.211	0.265
Range	3–96	3–84		
Postoperative complications, n (%)				
Wound infection	9 (0.3)	2 (0.4)	0.224	0.636
Hydrocele	40 (1.3)	8 (1.7)	0.472	0.492
Recurrence	21 (0.7)	13 (2.7)	18.460	<0.001*
Atrophy of testis	0	0	—	—
Epididymitis	3 (0.1)	1 (0.2)	0.47	0.493
Injury of vas deferens	0	1 (0.2)	6.51	0.011
Scrotal hematoma	2 (0.1)	1 (0.2)	1.309	0.308
Satisfaction, n (%)				
Very satisfied	2752 (89.1)	389 (81.7)	33.045	<0.001*
Somewhat satisfied	278 (9.0)	63 (13.2)		
Somewhat dissatisfied	53 (1.7)	18 (3.8)		
Very dissatisfied	6 (0.2)	6 (1.3)		

*Means statistically significant difference.

For the patients with infertility due to low semen quality, there was no statistical difference between the patients of MIMV and CMSV in natural pregnancy, improved sperm concentration, improved sperm motility, improved semen result, and semen results converted to normal (Table 3).

**Table 3:** Postoperative effectiveness for patients with low semen quality

Postoperative effectiveness	MIMV, n(%)	CMSV, n(%)	X^2^	P value
Achievement of natural pregnancy	1093 (52.2)	169 (51.2)	0.111	0.739
Improved semen results	1815 (86.7)	290 (87.9)	0.271	0.603
Improved sperm concentration	1667 (80.1)	261 (79.1)	0.047	0.828
Improved sperm motility	1053 (50.3)	172 (52.1)	0.384	0.535
Converted to normal semen results	1184 (56.6)	180 (54.5)	0.462	0.497

For patients with scrotal pain, we found no statistical difference between MIMV and CMSV in preoperative pain scores and pre-post difference of pain scores.

For the patients with persistent prostatitis, no statistical difference was found between MIMV and CMSV in pre-post difference of NIH-CPSI score (Table 4).

**Table 4:** Postoperative effectiveness for patients with scrotal pain and patients with persistent prostatitis

Postoperative effectiveness	MIMV	CMSV	t	P value
Patients with scrotal pain				
Pre-post difference of pain scores	3.3 ± 0.6	3.4 ± 0.8	0.896	0.394
Patients with persistent prostatitis				
Pre-post difference of NIH-CPSI score	14.1 ± 3.2	13.8 ± 3.1	1.476	0.145

For patients with varicocele in Grade 3 and no symptoms, varicocele disappeared in 44 patients (72.1%) after MIMV and in 5 patients (71.4%) after CMSV, with no statistical difference found (X^2^ = 0.002, P = 0.964).

For adolescents with testicular atrophy, symptoms disappeared in 31 patients’ (83.7%) after MIMV and in 3 patients (75.0%) after CMSV, with no statistical difference found (X^2^ = 0.197, P = 0.657).

## Discussion

In the course of microscope-assisted surgery, all testicular venous return routes can be observed directly, and it has been reported that this approach can significantly reduce the recurrence rate of varicocele^28^. Under the microscope allowing for 6 to 25 multiple magnifications of surgical field, the surgeons can perform meticulous hemostasis, identify and protect the testicular arteries and lymphatic vessels, and avoid intraoperative unintentional iatrogenic injury^29^. The only drawback of microscope-assisted surgery seems to be the longer operation time than that of traditional open inguinal surgery, thus requiring extensive training^30^. Microscope-assisted varicocelectomy can be performed using either the inguinal approach or the subinguinal approach, both of which can improve the surgeon’s identification and accuracy. From an anatomical perspective, there are more and thinner veins and testicular artery branches in subinguinal region than in inguinal region^8^, making it more challenging for the surgeons to protect the artery and to ligate the veins in subinguinal region.

In addition, our local infiltration anesthesia and nerve block anesthesia has some advantages over the field block anesthesia reported by Ross^31^: (1) our range of anesthesia was smaller and more precise; (2) we used less local anesthetic; (3) we added adrenaline to extend the anesthesia time; (4) we had a one-shot operation without additional dose.

Using the results of physical examination, color Doppler ultrasound and venography, a wide range of recurrence rates (0% to 45%) has been reported^32^. Hsieh *et al*. performed high inguinal loupe-assisted varicocelectomy in 254 cases, revealing a palpable recurrence rate of 3.1%^33^. In patients who underwent MIMV, the recurrence rate, as determined using results of physical examination and/or color Doppler ultrasound, was 0.7%. The procedure was performed at the internal ring level of the inguinal channel, enabling the surgeon to minimize the number of veins and artery branches encountered.

Comparing the results of varicocelectomy from different studies, Hsieh *et al*. demonstrated a significant increase in sperm concentration and sperm forward motility, as well as a 37% pregnancy rate^33^. The present study also showed an improved semen result in 1815 patients (86.7%), with 1184 patients’ (56.6%) converting to normal in sperm concentration and motility. Nevertheless, the present study still noted a pregnancy rate of 52.2%. Among the 34 patients with recurrence, we found 29 patients has left renal vein entrapment.

Our proposed MIMV approach, as an evolution of varicocelectomy, has the following advantages. Firstly, we use local anesthesia instead of general anesthesia or lumbar anesthesia for most cases in MIMV, which could not only reduce the operation duration, but also make the patients free from fasting before surgery and catheterization after surgery, allowing patients to return to normal activities after a few hours. Secondly, MIMV is inguinal, but CMSV is subinguinal. We can visualize the 2–8 spermatic veins in the inguinal region. The vein has a larger diameter, and is therefore easy to separate. The number of internal spermatic veins is obviously smaller than that of the external inguinal ring. Thirdly, the ilioinguinal/iliohypogastric nerve block was performed to ease the discomfort during the division and ligation of the spermatic cord, while the genitofemoral nerve block was performed to reduce the pain of the incision and scrotum. In addition, semen quality was improved in the follow up of 3 months, and the pregnancy rate after MIMV was 52.2%. Furthermore, the possibility of missing out a branch of testicular vein is lower in MIMV than in CMSV, as venous veins are thicker and less in inguinal region than in subinguinal region^17^. Additionally, the recurrence rate of MIMV is much less than that of CMSV. Finally, the patients with MIMV were more satisfied with the surgery experience and effect compared to those with CMSV.

The strengths of our study include high efficacy and precision of MIMV, with low bleeding, high satisfaction and less complications, and a large sample size. Our research also has some limitations. First, the surgical approach was chosen by the patients, which may lead to volunteer bias and a risk of adverse selection owing to the asymmetric information between doctors and patients. Although every effort was made to provide extensive information about MIMV and CMSV to the patients by various means, including conversation, books, pictures, and experience of other patients, it cannot be excluded that some patients may not fully understand it. Second, performing only 2 preoperative and 2 postoperative semen analyses may result in the problem known as “regression towards the mean”^15^. Third, being a single-centre study, our results may be prone to Berkson’ bias, despite including the same surgeons and the same surgical approaches in the same hospital. Finally, although the study was non-randomised, we provided a large number of patients with CMSV as the standard control. The same surgeons performed MIMV and CMSV in the same hospital, no statistical difference was found in age, indications of varicocelectomy, laterality, grade, time to follow up, preoperative diameters of varicose veins, and preoperative duration of reflux between the patients in the two groups.

In summary, MIMV was associated with a lower recurrence rate, shorter operating duration, less time to return to normal activity, higher satisfaction, similar effectiveness (natural pregnancy, semen results, pain scores, NIH-CPSI score) and rate of other complications compared with CMSV.

## Electronic supplementary material

Supplementary Information
