# Supplementary material for: Modified Inguinal Microscope-Assisted Varicocelectomy under Local Anesthesia: A Non-randomised Controlled Study of 3565 Cases
Source: Sci Rep. 2018 Feb 12;8:2800. doi: 10.1038/s41598-018-21313-0 (PMC5809367; doi:10.1038/s41598-018-21313-0)
Supplement: Supplementary file 1 — Supplementary Information [file 41598_2018_21313_MOESM1_ESM.pdf]

Modified Inguinal Microscope-Assisted Varicocelectomy under Local Anesthesia: A Non-randomised Controlled Study of 3565 cases

Jin Wang[1#], Qian Liu[1, 2#], Xun Wang[1], Rijian Guan[1], Sen Li[1], Youpeng Zhang[1], Yongbiao Cheng[1], Hanqing Zeng[1], Yong Tang[1*], Zhaohui Zhu[1*]

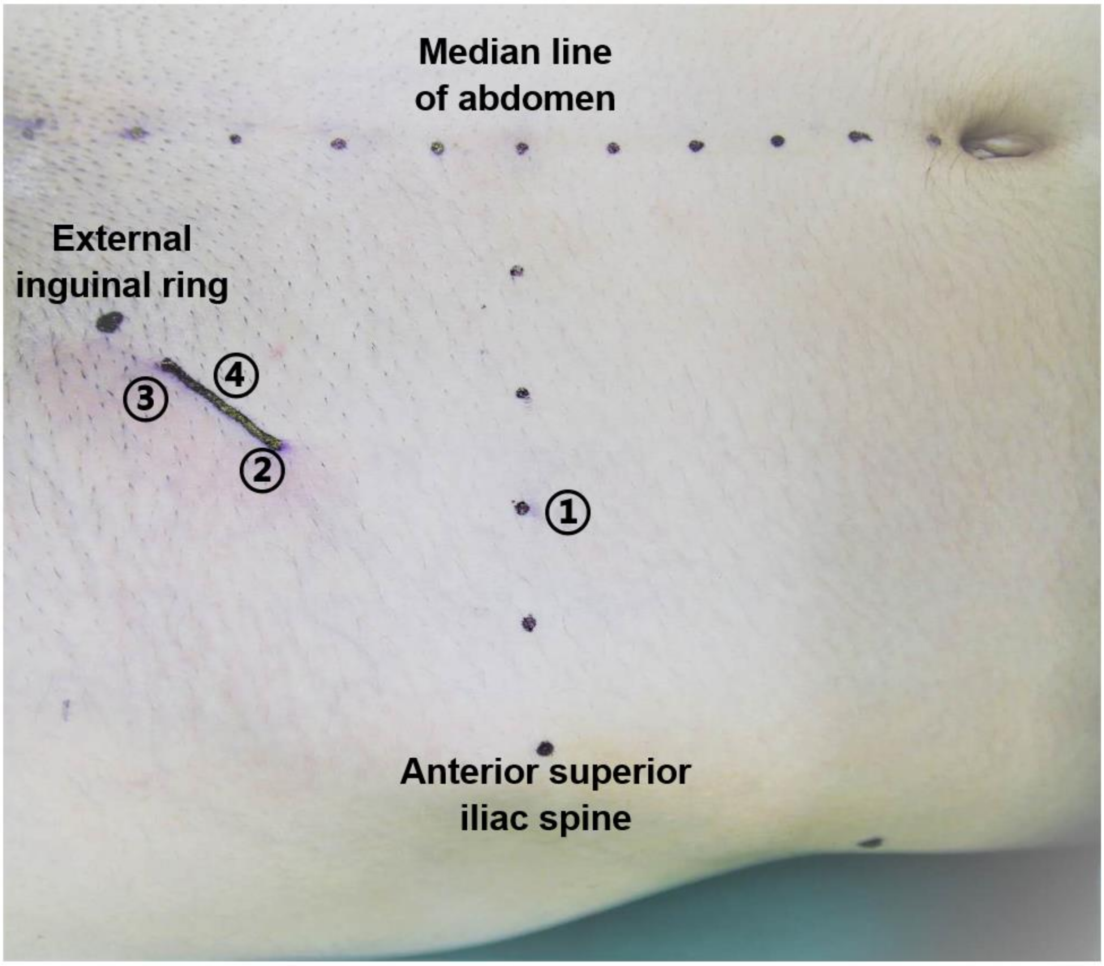

Supplementary Figure 1. The incision location was marked (A. external inguinal ring, B. a 2-cm marking line at the direction of iliac crest with 1 cm away from the external inguinal ring (① at the 2/5 outside of the vertical distance between ipsilateral anterior superior iliac spine and median line of abdomen, ② the upper margin of incision, ③the lower margin of incision, ④ the midpoint of the incision).

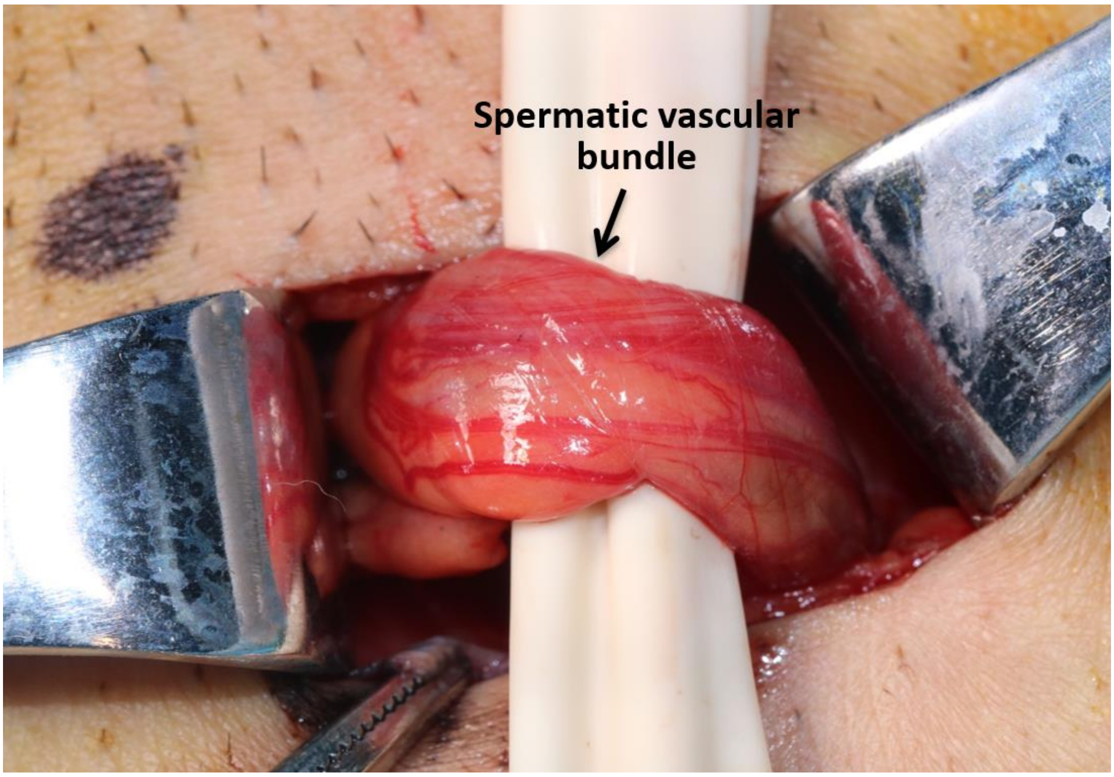

Supplementary Figure 2. The skin and subcutaneous tissue were incised, exposing the external oblique aponeurosis, and a longitudinal incision was made. Blunt dissection of the cremaster muscle was performed and the spermatic cord below the muscle was identified, facilitating the placement of the spermatic cord to the skin incision with a piece of rubber sheet.

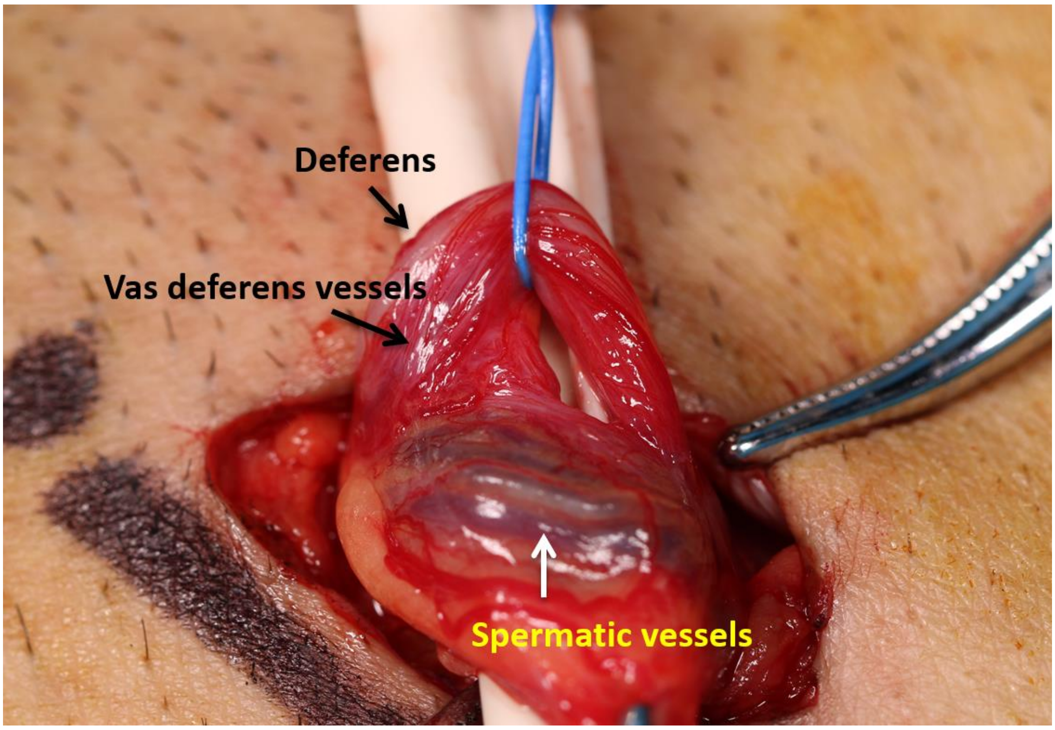

Supplementary Figure 3. The spermatic vascular bundle was then placed under a microscope with a resolution increase of 8-fold. The fascia of the spermatic cord was incised, and 1-2 larger spermatic veins were raised, to expose the remaining spermatic cord blood vessels and surrounding fat tissue, which were raised at the same time, visualizing the obvious boundaries with the vas deferens vascular system. Blunt dissection at the junction of the vas deferens vascular system was conducted.

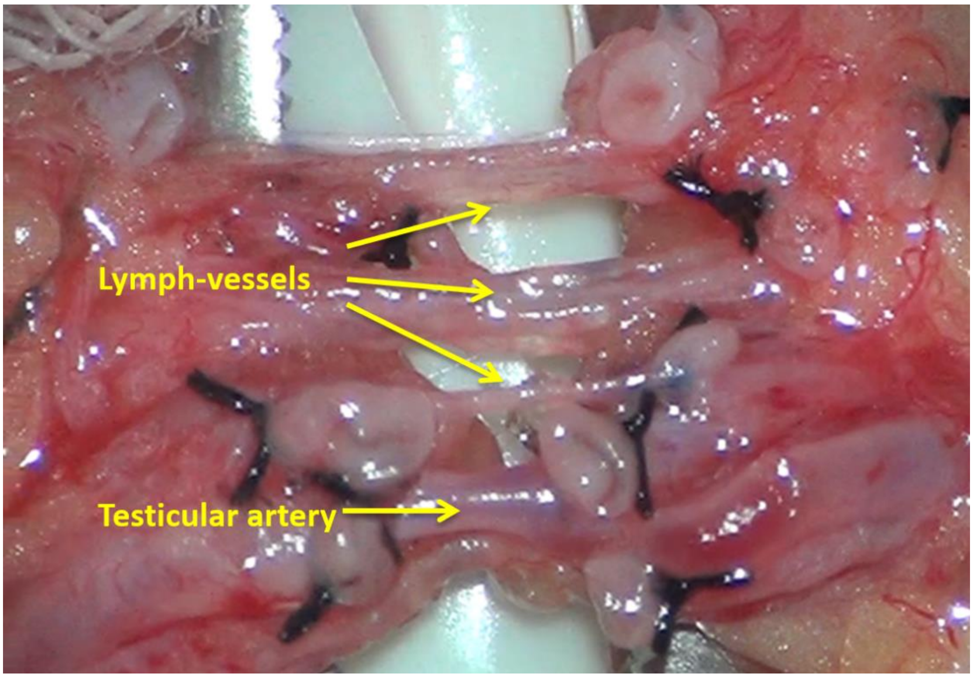

Supplementary Figure 4. The testicular artery and lymph vessels were carefully isolated. Vascular pulsation and blood flashing were observed or micro ultrasonic Doppler was used to confirm and protect the testicular artery. All the internal spermatic veins were ligated using Surgical Silk 5-0 and incised. Thick veins with no surrounding artery or lymph-vessel were ligated together.

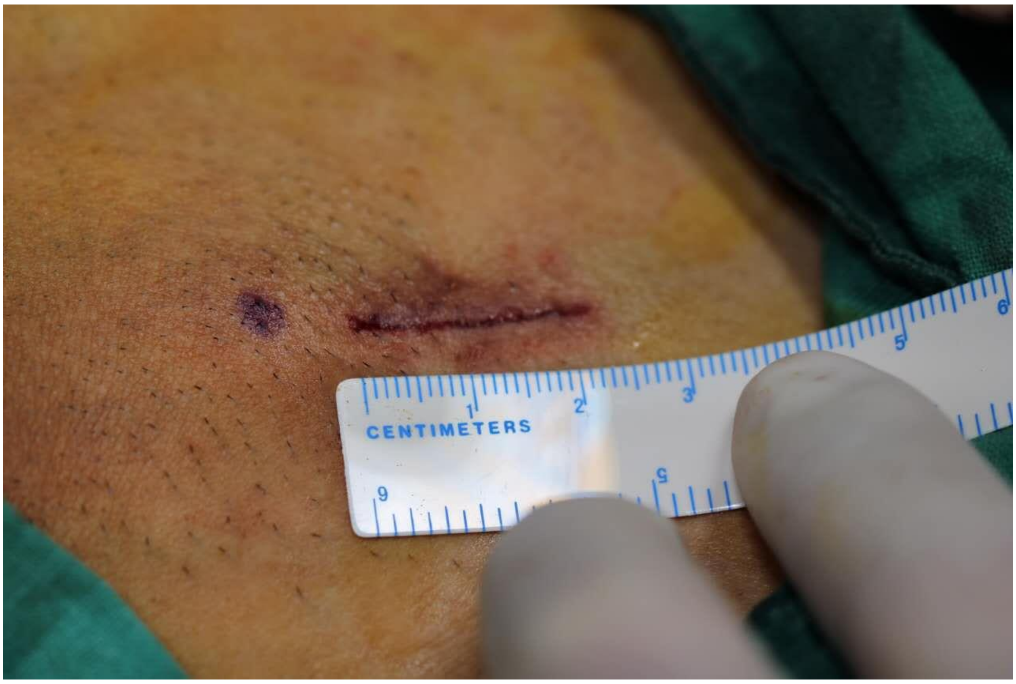

Supplementary Figure 5. Finally, a check for performed for obvious leakage and bleeding, sutured the fascia of the spermatic cord and the muscle of the testis, sutured each layer, and glued the incision.
